# CCR2 Inhibition Reduces Neurotoxic Microglia Activation Phenotype After Japanese Encephalitis Viral Infection

**DOI:** 10.3389/fncel.2020.00230

**Published:** 2020-08-13

**Authors:** Swati Singh, Gajendra Singh, Swasti Tiwari, Alok Kumar

**Affiliations:** Department of Molecular Medicine and Biotechnology, Sanjay Gandhi Postgraduate Institute of Medical Sciences (SGPGIMS), Lucknow, India

**Keywords:** japanese encephalitis virus infection, microglia activation, proinflammatory markers, CCR2 chemokine receptor, CCR2 chemokine receptor inhibition

## Abstract

Controlling the proinflammatory response of microglia by targeting chemokines (C-C motif) receptor 2 (CCR2) could be an important therapeutic approach for Japanese encephalitis virus (JEV) infection. Here, through JEV infection to BV2 microglia and young BALB/c mice, we investigated that CCR2 is highly upregulated after JEV infection and plays a key role in determining microglia activation phenotype and associated with neurotoxic proinflammatory mediators of TNF-α and IFNγ. In addition, we found JEV infection to BV2 microglia causes an increase in microglial proliferation and cell body area at day 1 and day 3. Using the agonist molecule of CCR2 inhibition; RS102895, significantly reduces microglia reactive phenotype and nitric oxide production. Further, to define the role of CCR2 in functional responses of microglia and their activation phenotype, we performed *in vitro* cell scratch functional assay and ImageJ analysis. When compared with control, microglia cells showed a significant increase in elongated or rod-like activated phenotype in JEV-infected cells at 24 h post-infection and CCR2 inhibition significantly reduced the elongated activation phenotype induced by JEV infection, suggesting that CCR2 acts as a critical regulator for microglia activation phenotype after JEV infection. We found that JEV-infected mice treated with RS102895 had less microglia activation and reduced mRNA expression of CCR2 and proinflammatory mediators such as IFN-γ in cortical tissue. Collectively, our data indicate that CCR2 drives reactive phenotype of microglia and its inhibition reduces microglia activation and neurotoxic proinflammatory mediators after JEV infection.

## Introduction

Recently, both experimental and clinical studies demonstrated that uncontrolled neuroinflammatory responses of central nervous system in Japanese encephalitis virus (JEV) infection is a major contributor to cell death and neurological dysfunction (Chen et al., 2012). JEV infection induced a neuroinflammatory response including microglia activation that further found engaged in release of proinflammatory mediators (e.g., TNF-α, IFN-γ) along with production of reactive oxygen species that contribute to neuronal cell death in bystander fashion (Das et al., 2008; Thongtan et al., 2010; Chen et al., 2012). However, the underlying molecular mechanism of dysregulated proinflammatory responses of microglia after JEV infection is not well understood. Though, in some studies, of microglia migration dynamics, alteration in their morphology including activation from resting phenotype, and adaptation of phagocytic morphologies are observed in association with their neuroinflammatory responses and neuronal cell death after JEV infection (Kreutzberg, 1996; Ghoshal et al., 2007; Kettenmann et al., 2011; Sips et al., 2012).

Chemokines are found to play an important role in recruitment of leukocytes and other immune cells in the specific area of the JEV-infected brain. For instance, chemokines (C-C motif) ligand-2 (CCL-2) and its receptor chemokines (C-C motif) 2 (CCR2) were found upregulated in brain cortex, striatum, thalamus, hippocampus, sub-ventricular zone, and midbrain area in JEV infection (Swarup et al., 2008; Das et al., 2011; Srivastava et al., 2012; Han et al., 2014) and associated with infiltration of monocytes, T-lymphocytes, and natural killer cells in inflamed brain areas (Rollins, 1991; Getts et al., 2008; Semple et al., 2010). CCR2 expression was also found to be significantly increased in resident immune cells of human microglia followed by JEV infection (Lannes et al., 2017), suggesting that CCR2 plays a key role in the regulation of microglia function and JEV pathogenesis. In recent study, it is also elucidated that CCR2 inhibition attenuates microglia activation and proinflammatory response to kainic acid (KA)-induced injury and reduces microglia-mediated neuronal cell death pathways (Tian et al., 2017). But, CCR2-dependent changes in microglia phenotype and their response followed by JEV infection need to be elucidated further. Here, we set out to investigate the cellular mechanism that drives proinflammatory response of microglia after JEV infection and to establish the role of CCR2 in JEV pathogenesis. The aims of the current study are: (1) to test the hypothesis that CCR2 activation is associated with proinflammatory response of microglia, and (2) to determine that systemic administration of CCR2 antagonist molecule RS102895 reduces proinflammatory response of microglia in JEV-infected mice.

## Materials and Methods

### Virus

An Indian neurovirulent, GP78 (GP 78668A) strain of JEV was used in the study. Virus was propagated in 2–3 weeks old suckling mice brain. A total of 25 μl from stock was inoculated intracerebrally in 2–3 weeks old suckling mice. After 4 days of infection; the mice were deeply anesthetized with chloroform, sacrificed, and brain tissue was homogenized in sterile phosphate-buffered saline (PBS) and virus titer was determined by the standard plaque assay (Yang et al., 2004).

### Animal

BALB/c mice 2–3 weeks old were used throughout the study. Mice were procured and housed at the animal care facility of SGPGIMS, Lucknow. Mice were fed with protein-rich diet and water *ad libitum*. The animals were maintained in an air-conditioned room (25 ± 2°C) with 12 h light (7:00–19:00) and dark cycle. All the experiments were performed during the daylight cycle.

### JEV Inoculation

BALB/c mice were inoculated with 3 × 10^6^ plaque-forming units (PFU) resuspended in 20 μl of PBS by using stereotaxic intracerebral injection with bregma and lambda on the same horizontal plane as described before Shukla et al. (2016). Control mice were inoculated with sterile 1× PBS (Sigma, USA). Mice were monitored daily and were sacrificed at days 3 and 7 post-inoculation. The brains were excised aseptically and were processed for biochemical analysis.

#### Study 1

Sham and JEV-infected mice (*n* = 4/group) were used for immunohistochemistry studies. At 7 days post-infection, mice were transcardially perfused with ice-cold 0.9% saline (100 ml), followed by 300 ml of 4% paraformaldehyde. Brains were removed and post-fixed in 4% paraformaldehyde overnight, and cryoprotected in 30% sucrose and were processed for microglia analysis.

#### Study 2

Sham and JEV-infected (*n* = 7/time point/group) mice were transcardially perfused with ice-cold 0.9% saline (100 ml) at 3 and 7 days post-infection. Ipsilateral cortical tissue was rapidly dissected and snap-frozen on liquid nitrogen for RNA extraction and western blotting.

#### Study 3

A CCR2 antagonist, RS102895, was intraperitoneally (i.p.) injected by giving a dose of 5 mg/kg at 72 h before JEV infection (*n* = 6) or control (*n* = 6) and once daily for the following 5 days. Mice were transcardially perfused with ice-cold 0.9% saline (100 ml), and ipsilateral cortical tissue was rapidly dissected and processed for snap-freezing on liquid nitrogen for RNA extraction and western blot analysis.

### BV2 Microglia Cell Culture, Cell Body Area, and Proliferation Analysis

BV2 microglia (murine microglial cell line) were grown on poly-L-lysine (Sigma–Aldrich) coated with or without coverslips in a 24-well plate and were maintained in Dulbecco’s modified Eagle’s medium (DMEM; Invitrogen, Carlsbad, CA, USA) supplemented with 10% fetal equine serum (HyClone, Logan, UT, USA) and 1% penicillin and streptomycin (Invitrogen) at 37°C with 5% CO_2_. Mock or JEV (at a multiplicity of infection of 10 TCID50/cell) infections were introduced for various time periods and cell body area, and proliferation analysis was performed as described before (Flora et al., 2019; Verdonk et al., 2016). Briefly, bright-field high-magnification images were acquired at constant light intensity and exposure. Images were exported to ImageJ software, where cell body area was analyzed using selection drawing tools. Similarly, for cell proliferation assay, BV2 microglia cells were treated with mock or JEV infection at 37°C with 5% CO_2_ for the time period of 3, 24, and 72 h. Cells were trypsinized and were incubated with equal mixture of trypan blue and viable cells were counted using a hemocytometer.

### RS102895 Treatment and Scratch Analysis

For CCR2 inhibitor treatment, cells were pre-treated with CCR2 antagonist, RS102895 (100 ng/ml), for 4 h before mock or JEV infection for various time periods and were processed for mRNA analysis and scratch experiment for the phenotypic and functional characterization. For the functional experiment, a cross-pattern scratch was introduced into the microglia cell culture and the activated number of microglia cells within the scratch area was counted at twelve fields per well. All experiments were repeated three times.

### Cell Viability Assay

Cell viability was determined using a tetrazolium salt 3-(4,5-dimethylthiazol-2-yl)-2,5-diphenyltetrazolium bromide (MTT; Millipore Sigma) colorimetric assay. BV2 microglia cells were incubated in 96-well plates in DMEM containing 10% fetal calf serum and 1% penicillin and streptomycin (40 U/ml and 40 mg/ml, respectively). Ten microliters of MTT at a final concentration of 0.5 mg/ml was added to each well. After 3 h incubation in 5% CO_2_ at 37°C, media was discarded and formazan crystals were dissolved by adding 100 ml of DMSO to each well. The absorbance was measured at 540 nm using an absorbance microplate reader and cell viability was expressed as a percentage of surviving cells compared with the control cells.

### Nitric Oxide Assay

Nitric oxide (NO) release into BV2 microglia condition media was assayed using a Greiss reagent assay (Invitrogen; G7921), as per the manufacturer’s instructions. NO concentration was calculated using standard curves generated from a nitrite stock, and results were expressed in micromoles.

### Real-Time PCR

Total RNA was extracted from snap-frozen samples using an RNeasy isolation kit (Qiagen, Valencia, CA, USA) with on-column DNase treatment (Qiagen). cDNA synthesis was performed on 1 μg of total RNA using a Verso cDNA RT kit (Thermo Scientific, Pittsburg, PA, USA); the protocols used were according to the manufacturer’s instructions. Real-time PCR was performed using an ABI 7500 Sequence Detection System (Applied Biosystems) in the presence of SYBR Green. Standard PCR conditions were used as prescribed in SYBR Green I core reagent protocol. PCR was performed using nucleotide primers of CD11b, CCR2, TNF-α, and IFNγ (obtained from Integrated DNA Technology, Coralville, IA, USA). Gene expression was calculated relative to the endogenous control sample (GAPDH) to determine relative expression values, using the 2^−ΔΔCt^ method (where Ct is the threshold cycle). All experiments were repeated three times.

### Quantification of Virus

Quantification of viral RNA from brain tissue homogenate was performed by using Geno Sen real-time RT-PCR kit for JEV (Genome Diagnostics). Analysis was performed on an ABI 7500 real-time PCR system (Applied Biosystems). Virus copy number in the samples were determined by using pre-quantified JEV-specific RNA standards with known copy numbers, provided with the kit.

### Western Blotting

Proteins from ipsilateral cortical tissue were extracted using RIPA buffer, equalized, and loaded onto 5–20% gradient gels for SDS-PAGE (Bio-Rad, Hercules, CA, USA). Proteins were transferred onto nitrocellulose membranes and then blocked overnight in 5% milk in 1× TBS containing 0.05% Tween-20 (TBS-T). The membrane was incubated in rabbit anti-IBA1 (1:1,000; BD Transduction Laboratories), mouse anti-caspase 12 (1:1,000; Cell Signaling Technology), mouse anti-ubiquitin (1:1,000; Cell Signaling Technology), mouse anti-phospho-H2AX (1:1,000; Cell Signaling Technology), and rabbit anti-GAPDH (1:2,000; Sigma) overnight at 4°C, then washed three times in TBS-T and incubated in appropriate HRP-conjugated secondary antibodies for 2 h at room temperature. Membranes were washed three times in TBS-T, and proteins were visualized using Super Signal West Dura Extended Duration Substrate (Thermo Scientific, Rockford, IL, USA). Chemiluminescence was captured using ChemiDoc XRS + System (Bio-Rad), and protein bands were quantified by densitometric analysis using Bio-Rad Molecular Imaging Software. The data are normalized with endogenous control of GAPDH and expressed in arbitrary units. All experiments were repeated three times.

### Lipid Peroxidation Assay

Lipid peroxidation (LPO) was measured in tissue homogenate by assaying the level of thiobarbituric acid reactive substances as an index of peroxidation of lipids by using the method of Ohkawa et al. (1979).

### Immunohistochemistry

Twenty-micrometer coronal brain sections were selected, and standard immunostaining techniques were employed as described before (Kumar et al., 2016). Briefly, sections were incubated primary antibody rabbit anti-IBA1 (1:1,000; BD Transduction Laboratories) overnight at 4°C, then washed three times in 1× PBS and incubated with biotinylated anti-rabbit IgG antibody (Vector Laboratories, Burlingame, CA, USA) for 2 h at room temperature and avidin–biotin–horseradish peroxidase solution (Vectastain elite ABC kit; Vector Laboratories) for 1 h and then reacted with 3,30-diaminobenzidine (Vector Laboratories) for color development. Images were acquired using a fluorescent Nikon Ti-E inverted microscope, at ×10 (Plan APO 10× NA 0.45) or ×20 (Plan APO 20× NA 0.75) magnification. Exposure times were kept constant for all sections in each experiment. All experiments were repeated three times.

### Statistical Analysis

Quantitative data were expressed as mean standard errors of the mean (SEM). RT-PCR relative expression, nitric oxide production, activated microglia cell number, microglia cell body area, and proliferation were analyzed by one-way analysis of variance (ANOVA), followed by *post hoc* adjustments using Student–Newman–Keuls test. Remaining data were analyzed using Student’s *t*-test. Statistical tests were performed using GraphPad Prism program V.5 for Windows (GraphPad Software, San Diego, CA, USA). A *p*-value < 0.05 was considered statistically significant.

## Results

### JEV Infection Induces Microglia Activation, CCR2 Expression, and Classical Proinflammatory Mediators of TNF-α and IFNγ

**Figure 1:**
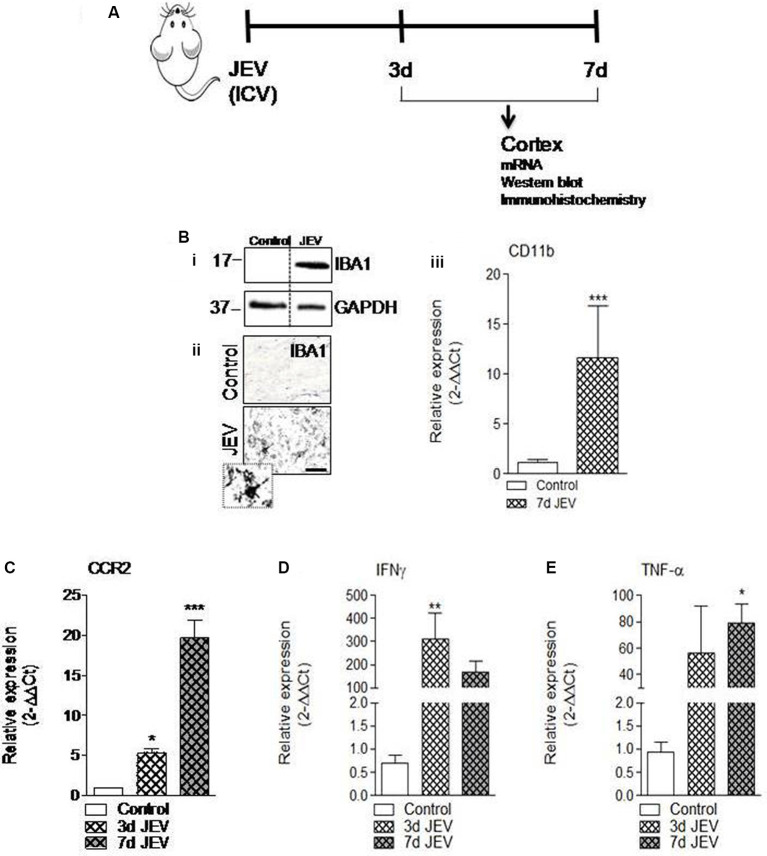
Microglia activation and its proinflammatory mediators significantly increases in cortex after Japanese encephalitis virus (JEV) infection. **(A)** JEV infection was intracerebrally (ICV) injected and tissue cortex were collected for mRNA, western blot, and immunohistochemistry analysis. **(Bi)** IBA1 protein expression at 7 days post-infection in the cortex tissue samples followed by JEV infection compared with control. **(ii)** Representative images for immunohistochemistry of Iba1 positive microglia cell at 7 days post-infection in cortex of JEV-infected tissue compared with control animals. Scale bars = 50 μm. **(iii)** qPCR analysis of microglia gene CD11b in the cortex of control and JEV-infected mice at 7 days post-infection. **(C–E)** JEV infection–induced expression of CCR2 and proinflammatory genes, TNF-α, and IFNγ in JEV-infected mice compared with control animals. One-way ANOVA; data = mean ± SEM; *n* = 3–7/group; *p < 0.05, **p < 0.01, and ***p < 0.001 vs. control group.

Consistent with previous finding (Wang and Deubel, 2011), we also noted that mRNA copy of JE viral infection significantly increases over time (1.4 × 10^5^, 4 × 10^5^, and 6 × 10^5^ copies/ml at 1, 3, and 7 days, respectively), which suggested that JE viral infection progresses over time. Moreover, increased microglia cell activation has been reported in the JEV-infected brain and found associated with neurological dysfunction and increased neurodegeneration (Chen et al., 2010). In the current study, we set out to investigate the mechanism that drives proinflammatory response of microglia activation after JEV infection and to establish the role of CCR2 in JEV pathology. For this, JEV infection was introduced into mice and cortical tissue was isolated at 3 and 7 days for mRNA, western blot, and immunohistochemistry analysis (Figure 1A). Our western blot analysis revealed that JEV infection robustly induced Iba1 protein expression in JEV-infected animals compared with control, suggesting that microglia activation significantly increases in JEV-infected cortical tissue (Figure 1Bi). On the basis of cell morphological analysis (as we have described in a previous publication Kumar et al., 2013), we further noted that hypertrophy-activated microglia, which is characterized by larger cell body area with thicker, shorter, and highly branched processes, significantly increases at day 7 in JEV-infected cortical tissue compared with control animals cortical tissue (Figure 1Bii). Next, our mRNA gene expression analysis of CCR2, CD11b, and classical proinflammatory markers of TNF-α and IFNγ suggests that JEV infection significantly increased mRNA level of CCR2 receptor, CD11b, TNF-α, and IFNγ when compared with control animals [*p* < 0.05 (TNF-α, CCR2 at 3 days), *p* < 0.01 (IFNγ at 3 days), *p* < 0.001 (CD11b at 7 days and CCR2 at 7 days); Figures 1Biii–E].

### JEV Infection Alters Microglia Phenotype, Increases Microglia Cell Body Area and Proliferation *in vitro*

**Figure 2:**
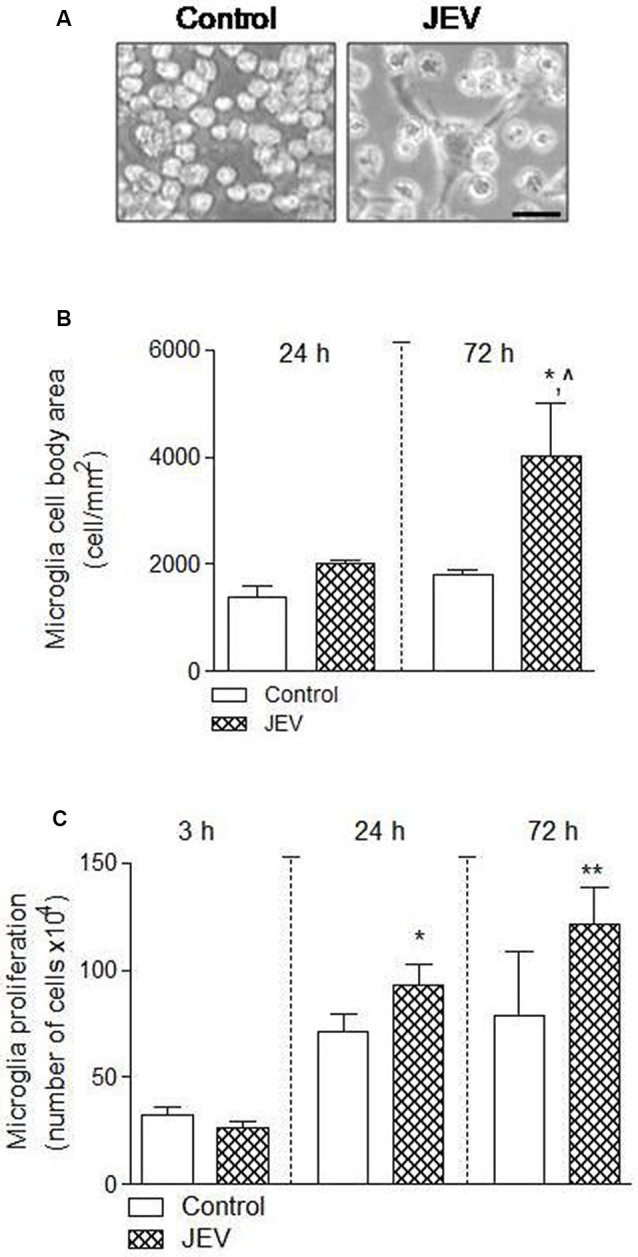
Microglia proliferation and cell body area increases after JEV infection compared with control cells. BV2 microglia cells were infected with JEV and microglia morphology was observed at different times of 24 and 72 h. **(A–C)** Microscopic morphometric analysis and microglia cell number analysis showed that microglia cell body area and proliferation significantly increases after JEV infection compared with control microglia cells. Data are expressed as mean ± SEM; *n* = 3–4/group; *p < 0.05, ^∧^*p* < 0.05 vs. control group at 72 h for microglia cell body area **(B)** and *p < 0.05 and **p < 0.01 vs. control group at 24 and 72 h for microglia proliferation **(C)**. One-way ANOVA.

The cell number and morphological analysis were performed using a hemocytometer and ImageJ software at different cell culture times of 3, 24, and 72 h in BV2 microglia after JEV infection and control cells. There was an increase in microglia cell body area [*p < 0.05 (when compared with 24 h control), ^∧^*p* < 0.05 (when compared with 72 h control); Figures 2A,B] and microglia cell number [*p < 0.05, **p < 0.01 (when compared with 3 h control); Figure 2C], suggesting that JEV infection increased activated microglia cellular phenotype and proliferation when compared with control microglia cells.

### CCR2 Inhibition Reduces Microglia Activation and Nitric Oxide Production in Microglia Cell Culture After JEV Infection

**Figure 3:**
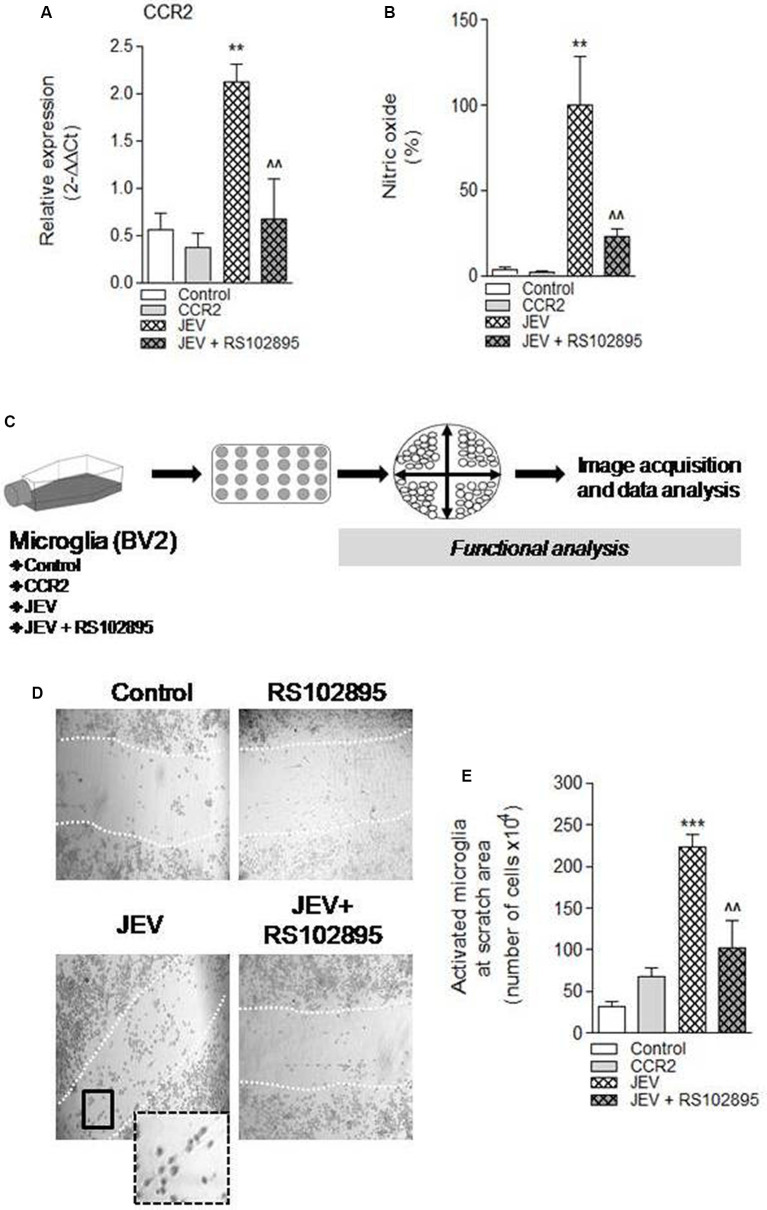
Chemokines (C-C motif) receptor 2 (CCR2) inhibition, by using molecular inhibitor RS102895, to BV2 microglia cell culture after JEV infection alters function response and reduces microglia activation phenotype. **(A)** Consistent with our JEV infection into mice model study, we further found that JEV infection–induced expression of CCR2 into BV2 microglia cell culture compared with control cells and RS102895 treatment significantly reduces its expression. **(B)** Similarly, nitric oxide (NO) analysis was also performed in supernatant of control, JEV-infected, and RS102895-treated JEV-infected microglia cells, and reduction in NO response was observed. Data are expressed as mean ± SEM; *n* = 3–5/group; **p < 0.01 vs. control and ^∧∧^*p* < 0.01 vs. JEV-infected microglia cells at 24 h. One-way ANOVA. **(C)** Further for the functional assays, microglia cells were seeded and cross-pattern scratch was performed after JEV infection and CCR2 treatment and migration analysis was performed. **(D,E)** After 24 h, images were acquired and were analyzed for their phenotypic analysis. At the scratch area, we observed that the elongated phenotype of microglia (activated) significantly reduces after CCR2 treatment compared with JEV-infected microglia cells and control microglia cells. Data are expressed as mean ± SEM; *n* = 3/group; ***p < 0.001 vs. control and ^∧∧^*p* < 0.01 vs. JEV-infected microglia cells at 24 h. One-way ANOVA.

CCR2 regulation was thought to regulate redox signaling and neuroinflammatory responses in CNS (Brune et al., 2013). Therefore, in the current study, we hypothesized that CCR2 drives neurotoxic microglia neuroinflammatory response after JEV infection. To test this hypothesis, we inhibited CCR2 by using CCR2 inhibitor, RS102895, in microglia cell culture of both JEV-infected microglia cell and control microglia cells, and analyzed nitric oxide production using a spectrophotometer and microglia activation phenotype. Note that *in vitro* study experiments, we used 100 ng/ml concentration of RS102895 after determining 100% of cell viability level for CCR2 and JEV infection to microglia cells. JEV infection significantly increases CCR2 expression and nitric oxide production in JEV-infected microglia cells when compared with levels in control microglia [**p < 0.01 (CCR2), **p < 0.01 (nitric oxide) vs. control microglia cells; Figures 3A,B]. In contrast, CCR2 treatment in JEV infection group resulted in a significant reduction of CCR2 expression and nitric oxide production [^∧∧^*p* < 0.01 (CCR2), ^∧∧^*p* < 0.01 (nitric oxide) vs. control microglia cells; Figures 3A,B], indicative of a reduced neurotoxic response of microglia in CCR2-treated JEV microglia when compared with control microglia cells. We then expanded our analysis to functional response of microglia and their activation phenotype analysis by performing *in vitro* scratch functional assay and ImageJ analysis as described before (Figure 3C). When compared with control microglia cells, there was a significant increase in elongated or rod-like activated phenotype of microglia in JEV-infected microglia cells at 24 h post-infection (*p* < 0.001 vs. control microglia cells; Figures 3D,E). Notably, CCR2 treatment significantly reduced the elongated activation phenotype induced by JEV infection (*p* < 0.01 vs. JEV-infected microglia cells; Figure 3E).

### CCR2 Inhibition Reduces Proinflammatory-Neurotoxic Mediators of Microglia After JEV Infection

**Figure 4:**
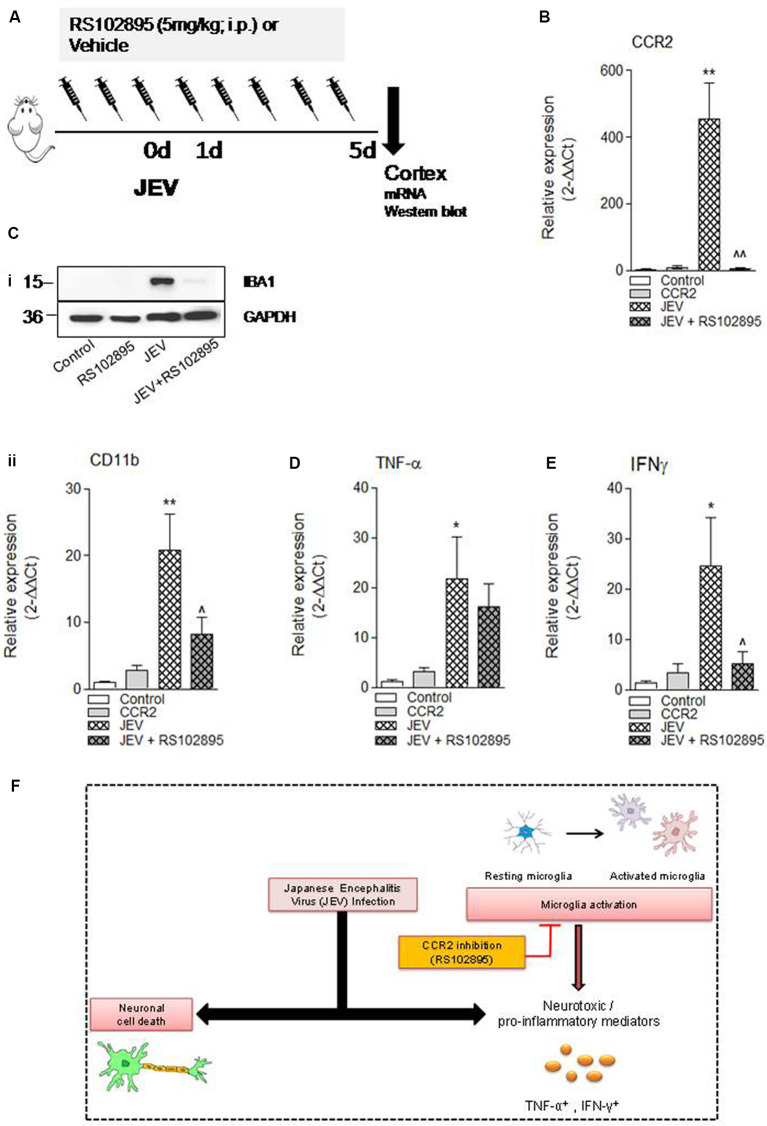
CCR2 inhibitor treatment reduces neuroinflammation and cell death markers after JEV infection. **(A)** RS102895 was intraperitoneally (i.p.) injected by giving a dose of 5 mg/kg at 72 h before JEV infection and once daily for the following 5 days. **(B–E)** mRNA and **(Ci)** protein western blot analyses were performed at 5 days post-JEV infection in JEV-infected and RS102895-treated JEV-infected animals. Data are expressed as mean ± SEM; *n* = 3–6/group; *p < 0.05 and **p < 0.01 vs. control group and ^∧^*p* < 0.05, ^∧∧^*p* < 0.01 vs. JEV-infected animals. One-way ANOVA. **(F)** Graphical abstract summarizing interaction of CCR2 with neuroinflammation after JEV infection.

To investigate the effect of CCR2 inhibition in JEV infection, we used CCR2 inhibitor RS102895 in JEV mice model as shown in Figure 4A. We assessed mRNA expression of CCR2, microglial CD11b expression, proinflammatory mediators of TNF-α, IFNγ, and neuronal cell death markers of LPO, poly-ubiquitin, H2AX, and caspase 12 in cortical tissue at day 5 in JEV-infected, RS102895-treated JEV-infected animals, and respective control animals to establish the effect of CCR2 on microglia activation and neuronal cell death pathways. As predicted, JEV infection significantly increases CCR2, CD11b, TNF-α, IFNγ, LPO, and poly-ubiquitin in JEV-infected cortical tissue when compared with levels in control tissue [**p < 0.01 (CCR2), **p < 0.01 (CD11b), *p < 0.05 (TNF-α), *p < 0.05 (IFNγ), *p < 0.05 (LPO); Figures 4B–E, Supplementary Figures S2A,B]. In contrast, CCR2 treatment in JEV-infected animals resulted in significantly reduced CCR2 and microglial CD11b mRNA expression along with proinflammatory marker of IFNγ expression at day 5 post-JEV infection, indicating reduced expression of CCR2 associated with reduced expression of proinflammatory neurotoxic mediators of microglia in the cortex of CCR2-treated JEV-infected mice when compared with JEV-infected mice alone [*p* < 0.01 (CCR2), *p* < 0.05 (CD11b, IFNγ); Figures 4B–E].

## Discussion

In the current study, we delineated the role of CCR2 in determining morphology of microglia and neuroinflammatory response in JEV pathogenesis. We found that post-JEV infection, CCR2 expression is significantly increased and associated with reactive phenotype of microglia and subsequent production of proinflammatory mediators of TNF-α and IFNγ. Furthermore, our results demonstrated that CCR2 inhibition is an important target for controlling reactive microglia morphology and neuroinflammatory response of JEV infection.

A number of preclinical and clinical human studies suggest that exaggerated neuroinflammatory response of microglia contributes to JEV pathologies (Ghoshal et al., 2007; Sips et al., 2012). However, limited information is available for the alteration of microglial morphological features after JEV infection. Under normal physiological conditions, microglia exist in resting phenotype (inactivated) and continuously screen the CNS microenvironment for the maintenance of homeostatic condition. In immune challenge condition of viral infection or injury to CNS, these microglia cells rapidly transform into an activated state, proliferate, migrate to challenge site, and participate in the presentation of antigens, phagocytosis, and promote the resolution process (Hanisch and Kettenmann, 2007; Ransohoff and Perry, 2009). However, in case of larger extent of immune challenge, microglia cell body transforms to amoeboid cell body which is characterized by lesser processes and large cell body, and adopt a very similar morphology to bloodborne macrophages (Hanisch and Kettenmann, 2007). Similarly, in our JEV study, we observed that microglia morphology transformed into a highly reactive phenotype and cell body area; proliferation largely increases after JEV infection compared with control microglia cells (Ghoshal et al., 2007). Moreover, by performing *in vitro* functional scratch analysis, we observed that microglia adopted elongated/rod-like morphology after JEV at scratch area compared with control microglia cells. We observed that these rod microglia cells have few and polar processes that is entirely polarized and have a narrow cell soma. Similarly, these changes have also been noted and found associated with infection such as typhus, syphilis, and sleeping sickness (Spielmeyer, 1922; Ziebell et al., 2012; Au and Ma, 2017). Along with this, we noted that CCR2 inhibitor treatment causes a decrease in activated/elongated morphology of microglia, suggesting that CCR2 chemokine signaling plays a key role in microglia morphology transformation and JEV-mediated pathogenesis. To determine the further role of this unique morphology in inflammatory response, we also evaluated the production of nitric oxide and found that CCR2 inhibition significantly decreases nitric oxide production in CCR2 inhibitor-treated JEV-infected microglia cells. The following findings are consistent with previous findings in which changes in microglia morphologies after viral infection are noted, and with treatment of anti-inflammatory molecules such as minocycline, activation morphology of microglia was found decreased (Mishra and Basu, 2008; Quick et al., 2017). However, the role and morphological attribute of elongated microglia by using a specific antibody, which discriminates these morphologies in JEV pathogenesis, needs to be explored further. Nevertheless, our findings represent that alteration in elongated microglia morphology is a general response rather than JE specific, although these findings highlight the fact that the following understanding about key pathologies can be helpful in controlling the immunopathological response against JEV.

In phagocytic cells such as microglia, CCR2 is a G protein-coupled seven-transmembrane spanning receptor (GPCR). CCR2 signals through these GPCRs, specifically Gi to activate extracellular signal-regulated kinase ERK1/2 signal pathways (Jimenez-Sainz et al., 2003). The increased expression of CCR2 is associated with increased infiltration of inflammatory monocytes NK and T cells at sites of inflammation, which are key components of proinflammatory cascade after JEV infection (Liu et al., 2018; Zhang et al., 2019). Microglia is a key player in neuroinflammation, and proinflammatory cytokine production responsible for progressive neuron damage and CCR2 on microglia has been implicated as a key player for proinflammatory response in many neurodegenerative diseases and thus important in regulating microglia-mediated neurotoxicity (Ghoshal et al., 2007; Terry et al., 2012; Kim et al., 2016; Chauhan et al., 2017; Käufer et al., 2018; Zhang et al., 2019). In the present study, we provide evidence that CCR2 plays a key role in strong inflammatory response and disease pathogenesis after JEV infection (Ghoshal et al., 2007). Furthermore, we used CCR2 antagonist molecule RS102895 to inhibit CCR2 expression during JEV infection both *in vivo* and *in vitro* and observed the key disease outcome measures so that JEV-induced brain pathologies can be understood. RS102895 has been previously used to interfere with CCR2 signaling in the brain (Hung et al., 2013) and also highlighted the block of enhancement of phosphorylation of NFkappaB (NF-kB/p65), and involved in modulation of LPS-mediated inflammation-induced pathologies (Cerri et al., 2016).

Increased level of CCR2 expression in our study is consistent with a previous finding in which upregulation of CCR2 level is reported in JEV infection (Chowdhury and Khan, 2017). Moreover, there is a direct relationship between increased CCR2 expression and proinflammatory response after JEV infection (Liu et al., 2018). We show increased CCR2 expression and classical proinflammatory markers of TNF-α and IFNγ in cortical tissue of JEV-infected animals. When we blocked CCR2 expression by using CCR2 inhibitor, proinflammatory markers such as IFNγ reduced (He et al., 2016), but there was no significant change observed in TNF-α compared with JEV-infected animals. In a previous study, it has been shown that mice deficient in CCL2 can only block 40–50% monocyte movement from bone marrow (Jia et al., 2008). This could be one of the reasons why CCR2 inhibitor treatment cannot do complete inhibition of inflammation during JEV infection. However further study for trafficking of CCR2^+^ inflammatory immune cells such as monocytes from bone marrow to the CNS, their interaction with microglia cells, functional response toward either protective or pathological role, and their inhibition by genetic knockdown such as by using siRNA needs to be explored further.

Finally, in our study, we aim to assess if CCR2 inhibitor treatment could be a potential target for reduction of JEV-induced cell death pathways. We investigated the impact of RS102895 on neuronal cell death after JEV infection. Surprisingly, we did not observe any significant reduction in neuronal cell death pathways upon RS102895 treatment except for a trend in decrease of cell death markers of lipid peroxidation (LPO) and poly-ubiquitin (Supplementary Figure S2A). The following results suggest that JEV infection triggers CCR2-independent neuronal cell death pathways, associated with bystander direct virus-mediated injury (Figure 4F). A potential caveat to the interpretation of the present neuronal cell death data is the lack of observation of cell death–related marker changes beyond 7 days as JE disease progressed to severity and the mice death rate significantly increased after JEV infection. Moreover, in our study, we could not include JE viral load analysis followed by CCR2 inhibitor treatment because of disease severity; however, a previous study had demonstrated that CCR2 deficiency in mice leads to decreased susceptibility against lethal infection of JEV, but there is no difference in viral load in the brain (Kim et al., 2016). However, we did perform key outcome measures such as changes in body weight and survival rate after JEV infection (data not shown) to ascertain the level of disease severity and determine whether CCR2 inhibitor treatment reduces the effect of post-JEV infection. We found that there is no change in body weight and survival rate in CCR2-treated JEV-infected brain at 7 days. On the contrary, a previous study of Liu et al. (2018), in which JEV infection was introduced through intraperitoneal injection, demonstrated that mice treated with CCR2 antagonists had a higher survival rate (about 60%); this also suggests that treatment of CCR2 inhibitor and disease outcome is also dependent on the loss of integrity of blood–brain barrier and severity of JEV infection.

Overall, the following study confirms the role of CCR2 in JEV pathogenesis. We have demonstrated that JEV infection causes an increase in the level of CCR2 and key neuroinflammatory mediators such as TNF-α and IFNγ, which reflects a deleterious effect and skewed microglia activation phenotype toward a more reactive phenotype. These observations highlighted the role of JEV-induced CCR2 activation in neuroinflammation. Nevertheless, further studies are required to affirm the biochemical mechanisms involved in the inflammatory regulation of CCR2 pathways as it is also possible that JEV infection could upregulate the CCR2 expression in astrocytes, infiltrating other immune cells such as neutrophils and leukocytes, and initiate the downstream cross-talk signaling cascades.

## Data Availability Statement

The raw data supporting the conclusions of this article will be made available by the authors, without undue reservation.

## Ethics Statement

The animal study was reviewed and approved by SGPGIMS institutional ethics committee.

## Author Contributions

AK: conceptualization, methodology, validation, formal analysis, investigation, data curation, visualization, and writing—original draft. SS and GS: methodology, data curation, and validation. ST: resources and supervision. All authors contributed to the article and approved the submitted version.

## Conflict of Interest

The authors declare that the research was conducted in the absence of any commercial or financial relationships that could be construed as a potential conflict of interest.
