# Supplementary material for: CCR2 Inhibition Reduces Neurotoxic Microglia Activation Phenotype After Japanese Encephalitis Viral Infection
Source: Front Cell Neurosci. 2020 Aug 13;14:230. doi: 10.3389/fncel.2020.00230 (PMC7439097; doi:10.3389/fncel.2020.00230)
Supplement: Supplementary file 1 [file Data_Sheet_1.PDF]

Table 1: Sequence primers and gene description

Gene symbol	Description	Size (bp)	Primer sequence
GAPDH	Glyceraldehyde 3-phosphate dehydrogenase	176	ATGACATCAAGAAGGTGGTG/ CATACCAGGAAATGAGCCTTG
TNF-α	Tumour necrosis factor alpha	164	CCATTCCTGAGTTCTGCAAAG/ GCAAATATAAATAGAGGGGGGC
IFNγ	Interferon gamma	388	GAAAGCCTAGAAAGTCTGAATAACT/ ATCAGCAGCGACTCCTTTTCCGCTT
CD11b or Itgam	CD11 antigen-like family member B	217	CCTTGTTCTCTTTGATGCAG/ GTGATGACAACTAGGATCTT
CCR2	C-C chemokine receptor type 2	73	TCATCCACGGCATACTATCAACA/ GTGGCCCCTTCATCAAGCT
Caspase 3	Caspase 3	422	AGGGGTCATTTATGGGACA/ TACACGGGATCTGTTTCTTTG
Caspase7	Caspase7	436	CAGACCGCTCCTCTATCATCT/ CATCGGTCTCCCCTAAAATG
