# Supplementary material for: CCR2 Inhibition Reduces Neurotoxic Microglia Activation Phenotype After Japanese Encephalitis Viral Infection
Source: Front Cell Neurosci. 2020 Aug 13;14:230. doi: 10.3389/fncel.2020.00230 (PMC7439097; doi:10.3389/fncel.2020.00230)
Supplement: Supplementary file 2 [file Data_Sheet_2.PDF]

Supplementary figures

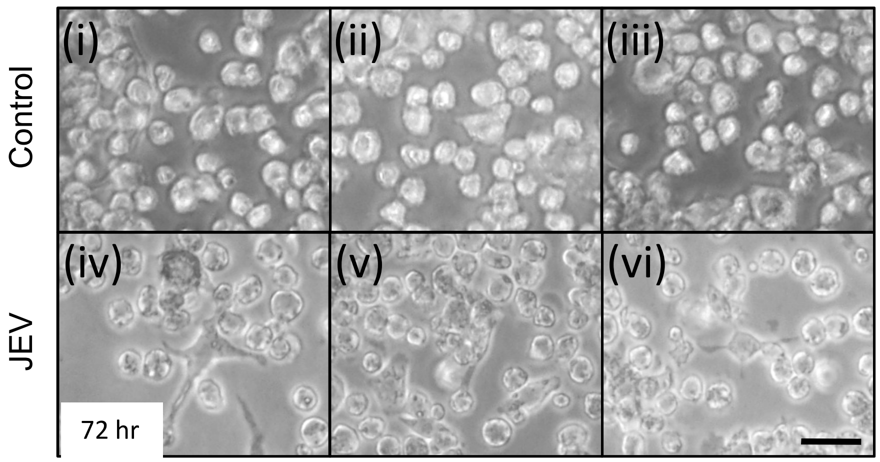

Figure S1. Microscopic images for cell proliferation analysis of control (i-iii) and JEV (iv-vi) infected microglia cells at 72 hr .

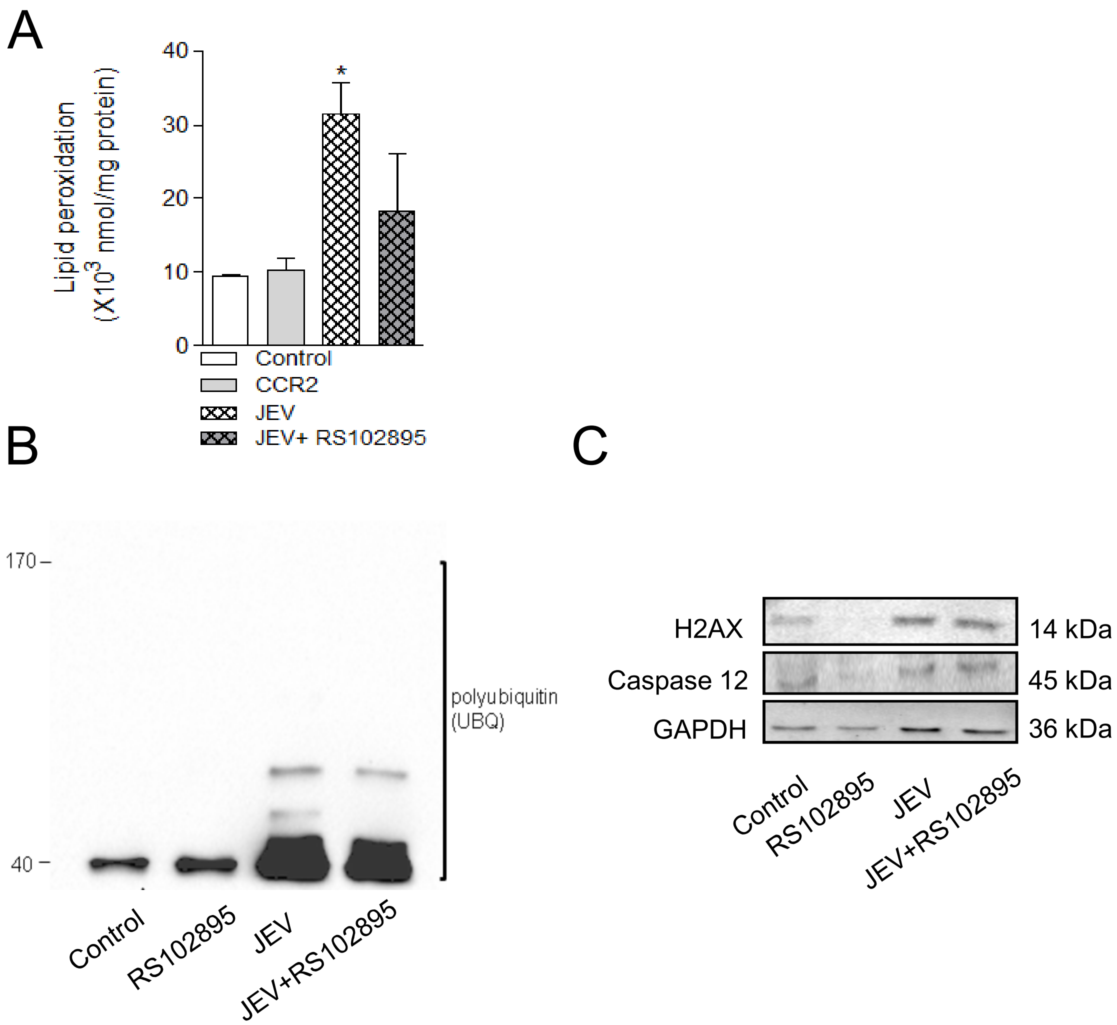

Figure S2: Inhibition of CCR2 by using its inhibitor RS102895 following JEV infection and protein expression of LPO (A), poly-ubiquitin (B) and H2Ax, Caspase 12 (C).
